# Using Buck's Fascia as an Integral Covering in Urethroplasty to Restore the Anatomical Structure of the Penis in One-Stage Hypospadias Repair: A Multicenter Chinese Study Comprising 1,386 Surgeries

**DOI:** 10.3389/fped.2021.695912

**Published:** 2021-08-09

**Authors:** Yin Zhang, Min Chao, Wei-ping Zhang, Yun-man Tang, Hai-chen Chen, Kai-ping Zhang, Ru-gang Lu, Xian-sheng Zhang, Dong-hua Lou

**Affiliations:** ^1^Department of Urology, Anhui Provincial Children's Hospital/Children's Hospital of Fudan University (Affiliated Anhui Branch), Hefei, China; ^2^Department of Pediatric Urology, Beijing Children's Hospital, Affiliated to the Capital Medical University, Beijing, China; ^3^Department of Pediatric Surgery of Children's Medical Center, Sichuan Academy of Medical Sciences and Sichuan Provincial People's Hospital, Affiliated Hospital of the University of Electronic Science and Technology of China, Chengdu, China; ^4^Department of Pediatric Surgery, Xiamen Maternal and Children's Health Hospital, Xiamen, China; ^5^Department of Urology, Nanjing Children's Hospital, Children's Hospital of Nanjing Medical University, Nanjing, China; ^6^Department of Urology, The First Affiliated Hospital of Anhui Medical University, Hefei, China; ^7^Department of Biostatistics, Nanjing Medical University, Nanjing, China

**Keywords:** hypospadias, penile anatomical structure, reconstructive surgical procedure, postoperative complications, multicenter study, Buck's fascia

## Abstract

**Objectives:** The objective of the study is to investigate the feasibility and efficacy of urethroplasty with a Buck's fascia integral-covering technique (BFIC) to wrap and restore the normal anatomical structure of the penis in one-stage hypospadias surgery.

**Methods:** One-stage surgeries for hypospadias management were performed using BFIC from January 2016 to September 2020 at four high-volume medical centers in China. The technique integrates Buck's fascia with glans wings to mobilize and wrap the urethra and restore penile anatomical relationships. The clinical data, postoperative follow-up data, and complications were recorded, and the results were analyzed.

**Results:** A total of 1,386 patients were included in the study: 1,260 cases of primary hypospadias and 126 cases of re-operations; distal in 382 cases (27.6%), mid-shaft in 639 (46.1%), proximal in 365 (26.3%); tubularized incised plate (TIP) in 748 cases, inlay-graft in 124, onlay-graft in 49, Mathieu in 28, free-tube graft urethroplasty in 406, and 31 of hybrid procedures. One thousand one hundred forty-two patients (82.4%) were found to have penile curvature (>10°) after artificial erection and all corrected by dorsal plication/s or transection of the urethra plate (UP) simultaneously. The median followed-up time was 27 months (6–62). A total of 143 (10.3%) complications were recorded: 114 (9.0%) in the primary operations and 29 (23%) in the re-operations, 15 (3.9%) in distal hypospadias, 61 (9.5%) in mid-shaft, and 67 (18.4%) in proximal. The complication rate in UP preservation and transection was 10.1 and 10.8%, respectively. Of all case complications, there were 73 (5.2%) of fistula, 10 (0.6%) of dehiscence, 22 (1.6%) of meatal stenosis, 21 (1.5%) of stricture, 6 (0.7%) of diverticulum, and resident curvature in 11 cases (1.2%). The overall complication rate in TIP and free-tube procedure was 9.8 and 9.9%, respectively, and fistula occurred in primary TIP of 33 cases (4.9%).

**Conclusions:** Buck's fascia with the glans can be used as an integral covering technique in one-stage distal to proximal hypospadias and primary or re-operative hypospadias repair. It is safe, feasible, and effective for the repair of hypospadias.

## Introduction

A survey of 27 European countries showed that the prevalence of hypospadias was 20.9 per 10,000 births and that the trend was increasing (1). Some studies have indicated a complication rate of ~10% for distal and over 50% for proximal hypospadias repairs (2). Many techniques have been described, and although no single, best method of urethroplasty has been clearly identified (3), investigators recently reported that beneficial modifications to hypospadias surgery could improve operative results (4, 5). It is currently accepted that any urethroplasty requires that some healthy vascular tissue be interposed between the urethroplasty and the skin (3, 6). Since 2016, there have been attempts to apply Buck's fascia (BF) and the glans as integral covering tissues, providing an intermediate layer to cover the neo-urethra in TIP and similar procedures in China. Differing results have been obtained in some centers, and the overall results showed that the effect was better than that of simple pedicled dartos fascia (DF). In the present study, we report the preliminary results from four large hypospadias treatment centers in China.

## Materials and Methods

### Patients

This was a retrospective analysis of clinical data from patients who underwent hypospadias repair with a Buck's fascia integral-covering (BFIC) technique from January 2016 to September 2020 at four high-volume medical centers in China—including the Anhui Provincial Children's Hospital (AHCH), the Beijing Children's Hospital (BJCH), the Sichuan Provincial People's Hospital (SCPH), and the Xiamen Maternal and Children's Health Hospital (XMCH). Each of the four centers has a senior pediatric urology surgeon who had at least 12 years of experience in hypospadias repair and performed all surgeries in each center.

The inclusion criteria were as follows: (1) primary or re-operation of hypospadias repair underwent one-stage urethroplasty and applied BFIC technique at the same time, and (2) BF was the only interposed barrier layer. The exclusion criteria were as follows: (1) two-stage hypospadias surgery; (2) without applying the BFIC technique; (3) applied other tissues as additional barrier to the urethra; and (4) with postoperative follow-up shorter than 6 months.

### Surgical Techniques

The crux of BFIC is to distinguish and mobilize the BF from the DF and tunica albuginea (TA), and keep the integrity and continuity of BF with the lateral glans wings.

**Figure 1:**
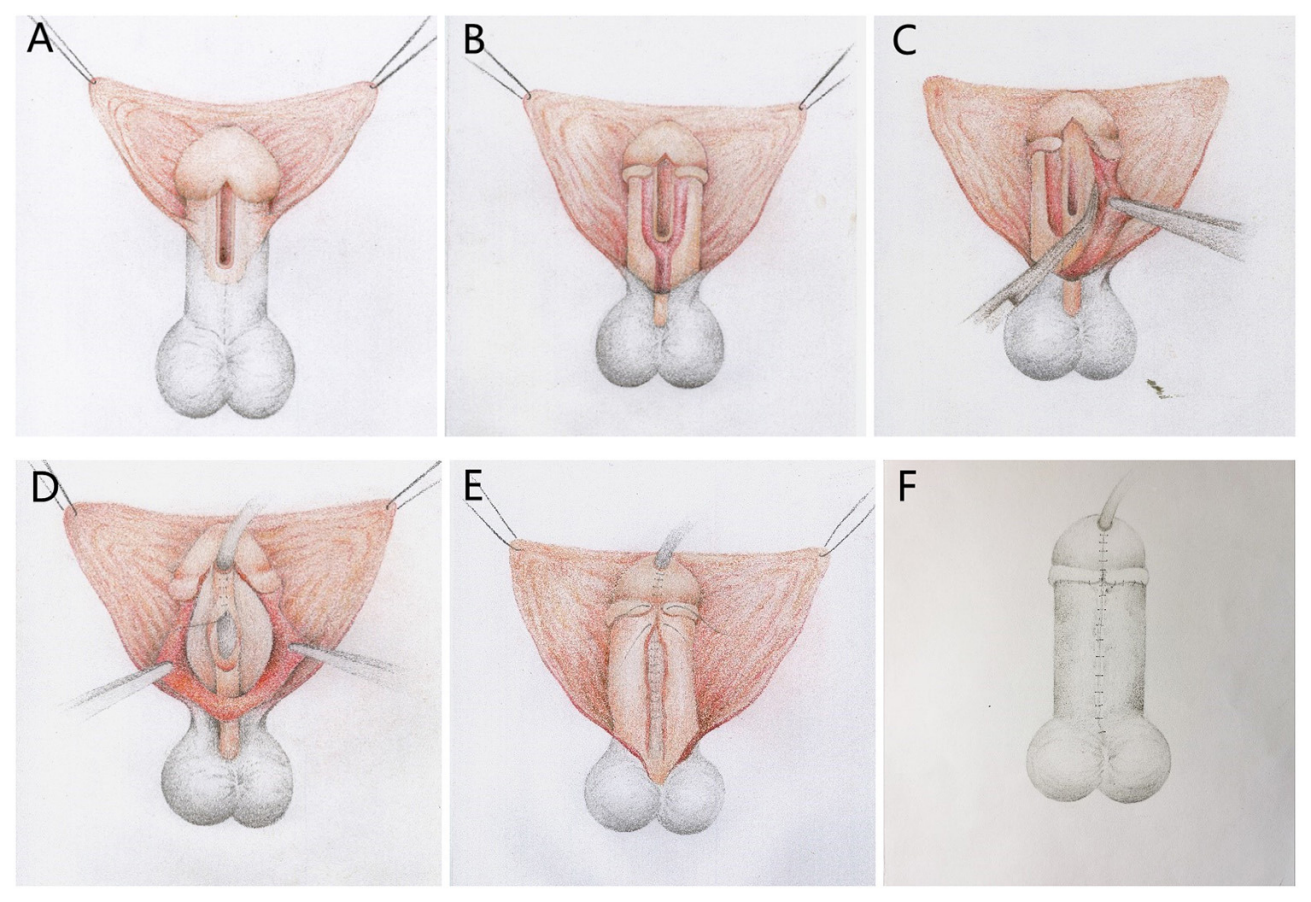
Diagrammatic sketch depicting the steps of our Buck's fascia integral-covering (BFIC) technique used to restore the normal anatomical structure of the penis with a TIP procedure. (**A**) Proposed incision lines. (**B**) Degloving procedure. (**C**) Buck's fascia combined with the glans wings was dissociated at the surface of the tunica albuginea from each side of the urethral plate to 2 and 10 o'clock positions with respect to the corpus cavernosum. (**D**) Urethroplasty. (**E**) Glansplasty, longitudinal closure of the Buck's fascia and the glans wings covering the urethra. (**F**) Restoration of the appearance of the penis.

**Figure 2:**
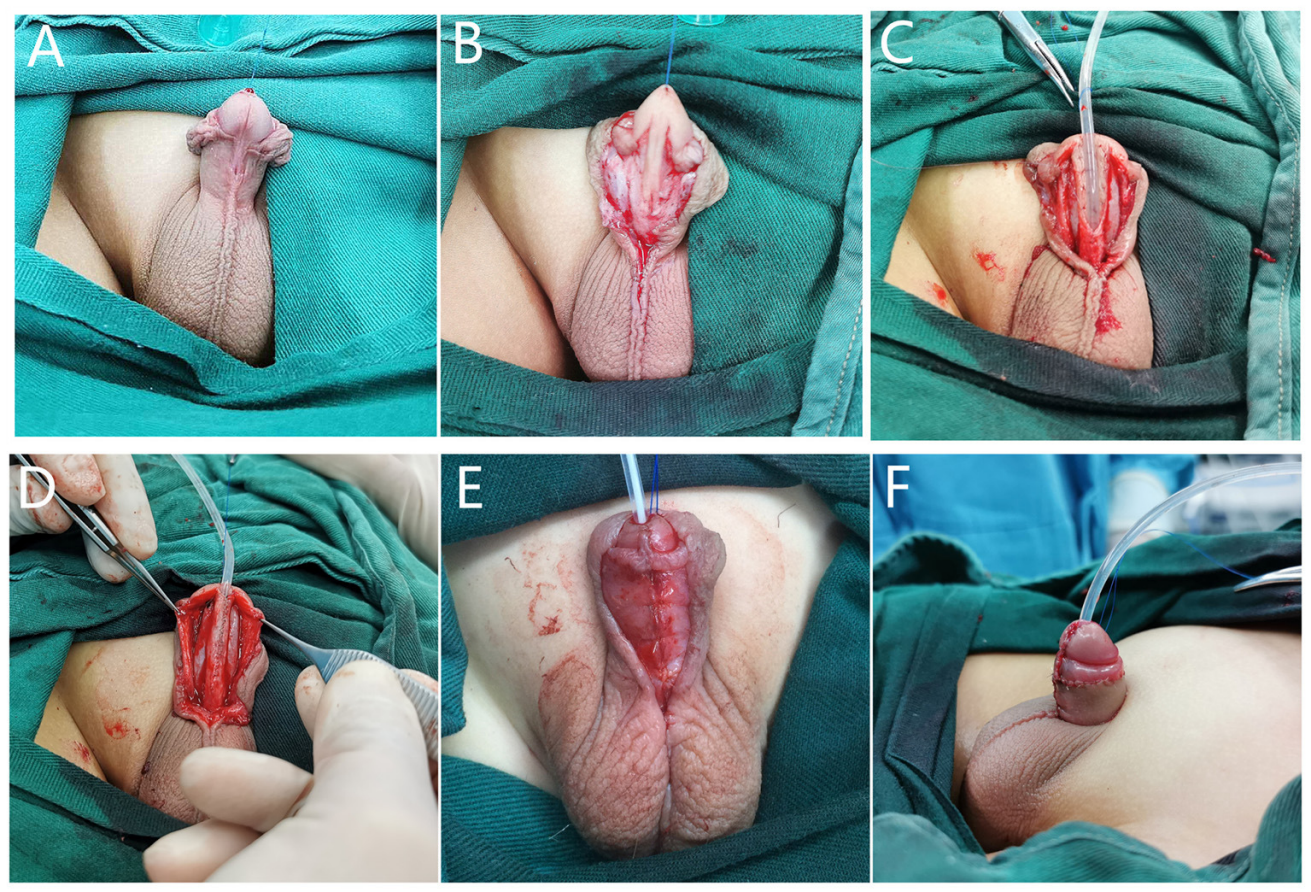
BFIC in inlay-graft procedure. (**A,B**) The urethral groove was shallow and the urethral plate was dysplastic after degloving. (**C**) We dissociated the Buck's fascia and glans wings over the tunica albuginea, and the free graft was laid in the center of the urethral plate. (**D**) The glans wing was developed on both sides with combined Buck's fascia intact. (**E**) After urethroplasty, we restored the normal anatomical structure of the penis with BFIC. (**F**) The appearance after surgery.

**Figure 3:**
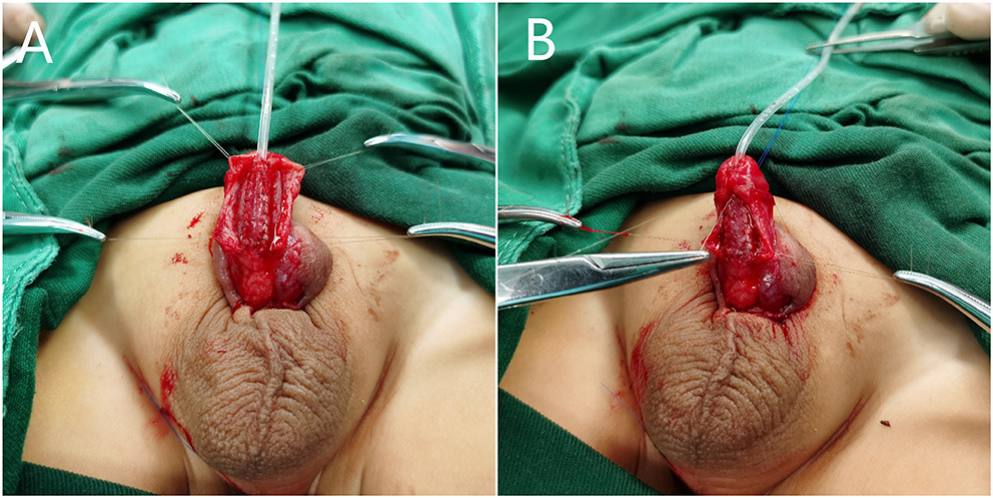
BFIC in onlay-graft procedure. (**A**) We dissociated the Buck's fascia and glans wings over the tunica albuginea, and the free graft was laid above the urethral plate. (**B**) After urethroplasty, we restored the normal anatomical structure of the penis with BFIC.

Taking the TIP procedure as an example, using BFIC in distal and mid-shaft hypospadias or proximal cases whose ventral curvature (VC) were easy to correct and the UP could be preserved, the key steps are shown in Figure 1. An incision is made below the corona on the dorsal surface. Ventrally, a U-shaped incision is made close to the UP. Within the glans, the incisions are made along the true urethral plate and parallel to each other. The penis is degloved, and then the incisions are deepened to the surface of the TA, then dissociated, and the BF is mobilized combined with the lateral glans wings from the surface of the TA to 2 and 10 o'clock of the corpus, making it approximately covering the neourethra ventrally without tension. After the mobilization of the lateral BF and the glans wings, artificial erection was performed. For 10° < VC < 15°, a midline dorsal plication was applied. For 15° ≤ VC < 30°, bilateral dorsal plications were accomplished. If we observed persistent VC ≥ 30°, we transected the UP, and concurrent dorsal plications were applied, and artificial erection was repeated; procedures such as free tube-graft will be considered. Then, the UP is incised in the midline, and two-layer UP tubularization is applied by using an interrupted 7-0 Vicryl suture. After urethroplasty, the BF and the glans wings were approximated as an intermediate layer to wrap the neourethra. Finally, the skin incisions were closed with superficial fascia beneath it to restore the appearance of the penis. According to the experience of the surgeon, surgical procedures such as inlay graft (Figure 2), onlay graft (Figure 3), or Mathieu can be used to augment the plate of inadequate-quality UP patients.

**Figure 4:**
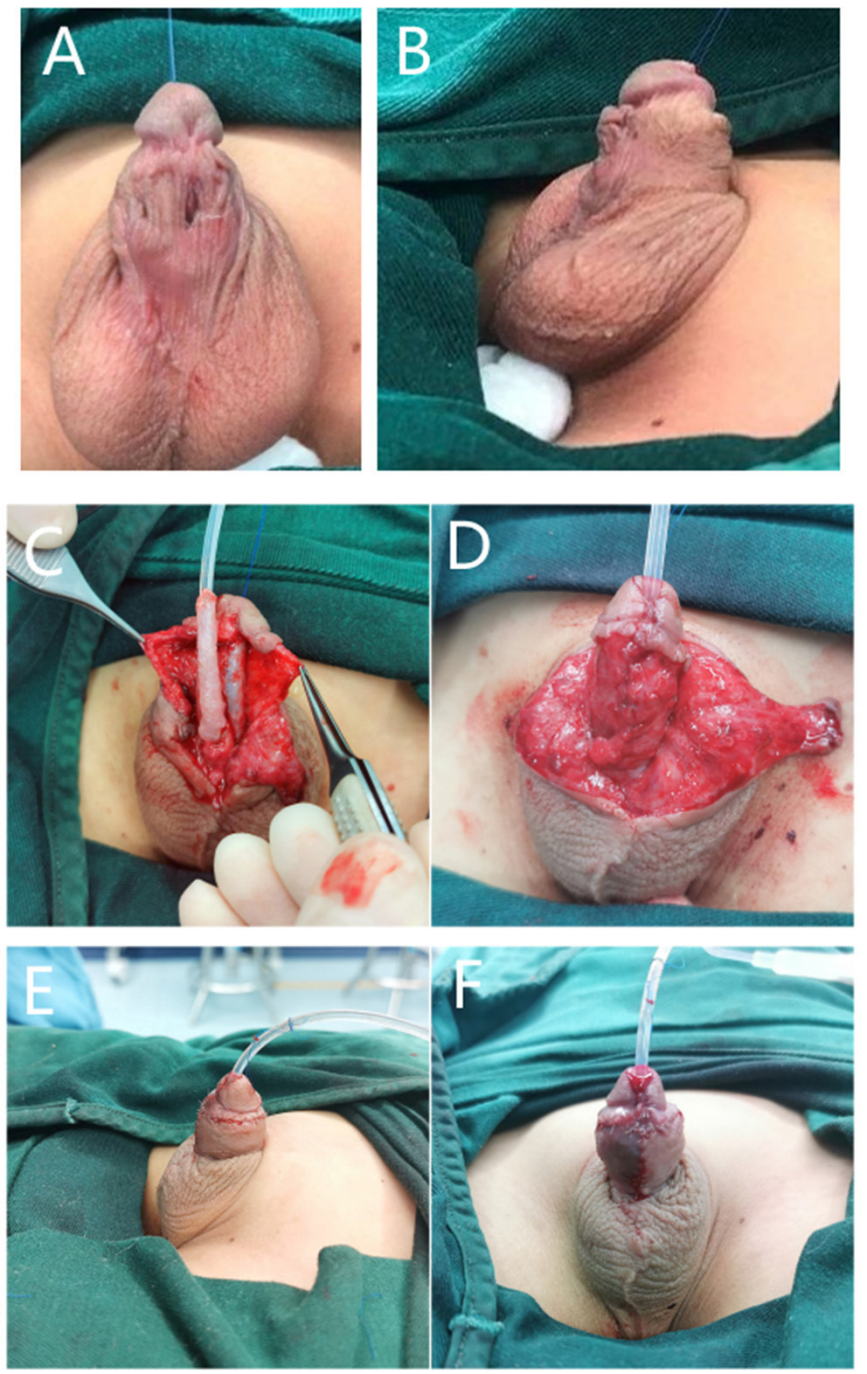
BFIC in a free-tube graft urethroplasty procedure. (**A,B**) A 7-year-old boy who underwent one surgery for hypospadias for which the preoperative condition was unknown, with residual VC, urethral fistula, and stricture. (**C**) We dissociated Buck's fascia and the glans wings over the tunica albuginea, corrected the VC, and completed the urethroplasty with a buccal mucosal graft tube. (**D**) Restoration of the normal anatomical structure of the penis with BFIC. (**E,F**) Frontal and lateral postoperative appearance.

For proximal hypospadias with severe VC after degloving, a free preputial tube-graft procedure was used in the primary surgeries, and a buccal mucosal graft tube (Figure 4) or hybrid procedure, such as free-tube combined with onlay or Duplay, was performed in proximal or re-operative cases. In free-tube procedures, a free graft was harvested from the dorsal prepuce or the buccal mucosa that was at least 15–20-mm wide and was longer than required to reconstruct the urethra.

### Follow-Up and Data Analysis

Sixty percent of the patients were followed by outpatient service, 30% by network platforms such as the mobile app “WeChat,” and 10% by telephone and email. At each center, surgeons involved in the study were responsible for collecting the data. The number of cases, operative procedures, age, and correction of curvature were recorded; complications such as urinary fistula, urethral dehiscence, urethral stricture, diverticulum, and residual curvature of the penis were noted. Cosmetic outcomes and uroflow data were incomplete in all centers, which were not included in the study.

## Results

A total of 1,386 patients at four centers, with a median age of 29.1 months (range: 6–180), underwent one-stage hypospadias surgery with the BFIC technique: 179 at AHCH, 62 at BJCH, 235 at SCPH, and 910 at XMCH; distal hypospadias in 382 cases (27.6%), mid-shaft in 639 cases (46.1%), and proximal in 365 cases (26.3%). Primary operations were performed in 1,260 cases and re-operations in 126 cases. In the primary/re-operative operations, 675/73 were done by TIP, 109/15 by inlay-graft, 39/10 by onlay-graft, 23/5 by Mathieu, 384/22 by free-tube graft, and 30/1 by other hybrid procedures. Hybrid procedures were retrieved only at XMCH, according to individual differences, including tube–onlay or tube–Duplay, etc. Urethroplasty with UP preservation and transection was 949 cases (68.5%) and 437 cases (31.5%), respectively. A total of 1,142 patients (82.4%) were found to have penile curvature during operation after artificial erection and all corrected by dorsal plication/s or combination with transection of the UP. Details are shown in Table 1.

**Table 1:** Clinical data and surgical procedures used at four medical centers, n/all (%).

	**Total** ** 1,386 cases**	**AHCH** ** 179 cases**	**BJCH** ** 62 cases**	**SCPH** ** 235 cases**	**XMCH** ** 910 cases**
Age (months, median with range)	29 (6–180)	42 (6–144)	21 (11–102)	48 (6–180)	22 (6–120)
Follow-up time (months, median with range)	27 (6–62)	27 (10–62)	19 (6–56)	24 (6–62)	32 (6–62)
Distal	382 (27.6%)	63 (51 & 12)	38 (38 & 0)	33 (3 & 30)	248 (239 & 9)
Mid-shaft	639 (46.1%)	92 (79 & 13)	24 (24 & 0)	193 (173 & 20)	330 (320 & 10)
Proximal	365 (26.3%)	24 (21 & 3)	0	9 (0 & 9)	332 (312 & 20)
Urethra plate (UP) preservation/transection	949/437	166/13	62/0	235/0	486/424
Primary/re-operation (cases)	1,260/126	151/28	62/0	176/59	871/39
Tubularized incised plate (TIP)	748 (675 & 73)	112 (98 & 14)	62 (62 & 0)	181 (134 & 47)	393 (381 & 12)
Inlay graft	124 (109 & 15)	38 (33 & 5)	0	30 (24 & 6)	56 (52 & 4)
Onlay graft	49 (39 & 10)	9 (7 & 2)	0	24 (18 & 6)	16 (14 & 2)
Mathieu	28 (23 & 5)	7 (3 & 4)	0	0	21 (20 & 1)
Free-tube graft	406 (384 & 22)	13 (10 & 3)	0	0	393 (374 & 19)
Hybrid procedure	31 (30 & 1)	0	0	0	31 (30 & 1)
Correction of ventral curvature (VC) during operation	1,142 (82.4%)	116 (64.8%)	25 (40.3%)	144 (61.3%)	857 (94.2%)

*Note. “&,” indicates cases of primary operation & re-operation. AHCH, the Anhui Provincial Children's Hospital; BJCH, the Beijing Children's Hospital; SCPH, the Sichuan Provincial People's Hospital; XMCH, the Xiamen Maternal & Children's Health Hospital*.

Complication data are shown in Table 2, with a median follow-up time of 27 months (range: 6–62). Complications occurred in 143 of the 1,386 patients (10.3%): 114 cases among the 1,260 primary operations (9.0%) and 29 cases among the 126 re-operations (23%), 15 cases (3.9%) in distal hypospadias, 61 cases (9.5%) in mid-shaft, and 67 cases (18.4%) in proximal. Complications in the UP-preserved patients were 96 cases (10.1%) and 47 cases (10.8%) occurring in the UP-transected patients. Of all the case complications, there were 73 cases of fistulas (5.2%), 10 of dehiscence (0.6%), 22 of meatal stenosis (1.6%), 21 of stricture (1.5%), and 6 of diverticulum (0.7%). Recurrent VC in 11 cases (1.2%) all occurred in the primary proximal hypospadias operations.

**Table 2:** Types of complications and incidence in distal, mid-shaft, proximal, primary, and re-operations, n/all (%).

**Complications** ** (cases)**	**Distal**	**Mid-shaft**	**Proximal**	**UP** ** preserved**	**UP** ** transected**	**Primary**	**Re-operation**	**Total**
Fistula	11	37	25	59	14	57 (4.5%)	16 (12.7%)	73 (5.2%)
Dehiscence	1	5	4	9	1	8 (0.6%)	2 (1.6%)	10 (0.7%)
Meatal stenosis	2	13	7	19	3	17 (1.3%)	5 (4.0%)	22 (1.6%)
Stricture	1	6	14	9	12	15 (1.2%)	6 (4.8%)	21 (1.5%)
Diverticulum	0	0	6	0	6	6 (0.5%)	0	6 (0.4%)
Recurrent VC	0	0	11	0	11	11 (0.9%)	0	11 (0.7%)
Overall complications	15/382 (3.9%)	61/639 (9.5%)	67/365 (18.4%)	96/949 (10.1%)	47/437 (10.8%)	114/1,260 (9.0%)	29/126 (23.0%)	143/1,368 (10.3%)

TIP was the only procedure carried out at the four centers; the overall complication rate in TIP was 9.8%. In the primary TIP procedure, complications occurred in 56 cases (8.3%) and 17 (23.3%) in re-operations. Fistula occurred in the primary and re-operation TIP procedure in 33 cases (4.9%) and 8 cases (10.9%), respectively. Free-tube graft operations were performed at two centers, 13 cases in AHCH and 393 cases in XMCH; the overall complication rate was 9.9%, 9.1% in primary operations and 22.7% in re-operations (Table 3).

**Table 3:** Fistula and complications rate in TIP and free-tube graft urethroplasty, n/all (%).

	**Fistula in** ** TIP**	**Complications in ** **TIP**	**Complications in** ** free-tube graft**
Primary	33/675 (4.9%)	56/675 (8.3%)	35/384 (9.1%)
Re-operation	8/73 (10.9%)	17/73 (23.3%)	5/22 (22.7%)
Overall incidence	41/748 (5.5%)	73/748 (9.8%)	40/406 (9.9%)

## Discussion

In a review of the literature concerning the incidence of complications of hypospadias, Wilkinson et al. (7) reported a 17.5% and 25% incidence of complications in high-volume and low-volume centers in the UK, respectively. Schneuer et al. (8) reported an overall postoperative complication rate of 13% in Australia. Spinoit et al. (9) depicted a complication rate of ~24.1% with long-term follow-up after primary hypospadias surgery in Belgium. Snodgrass et al. (10) described a 12% complication rate after follow-up in 792 cases of primary repair of hypospadias. Therefore, the occurrence of postoperative complications of hypospadias remains a great challenge for clinicians. Since the beginning of this century, many centers have promoted the use of the corpus spongiosum (11–13), along with the DF or tunica vaginalis fascia (TVF) as neourethral coverings in order to achieve improved results with urethral cutaneous fistula prevention (14). This technique has over the past two decades—from implementation to introspection—underwent processes of popularization, downturn, and central differentiation. It is suggested that the choice of which type of outer-layer covering method or material to use still correlates significantly with the experience of the surgeon and the specific treatment methods (15, 16).

There is an accepted concept that any urethroplasty should have some healthy vascular tissue interposed between the urethroplasty and the skin; most surgeons take tissue interposition during hypospadias repair as a routine step and often minimizes the incidence of urethrocutaneous fistula, especially for TIP procedures (6, 7, 10, 17). To date, DF, TVF, and other soft tissues, including spongious tissue, pedicled external spermatic fascia, adipose tissue from the scrotum, have been used to cover the neourethra (14–16), and TIP repair using TVF reduces the fistula rate and is superior to DF as a covering layer for primary TIP repair in midshaft hypospadias (18, 19). Demir described a new approach using a double-layer flap in TIP technique and found it beneficial for children and young adults preventing fistula as well as aesthetic appearance (20). Cimador et al. (21) even considered the use of a double ventral dartos flap, which should represent the first-line technique for coverage of distal urethroplasty in hypospadias repair with a lower complication rate. However, the findings of Thomas et al. (22), showed that the use of dartos flaps in hypospadias showed no statistically significant advantage over flapless repair for the fistula rates. Notably, there are a few large cohort sizes from multicenter studies on these coverage technologies, and future studies are required to confirm the final result.

**Figure 5:**
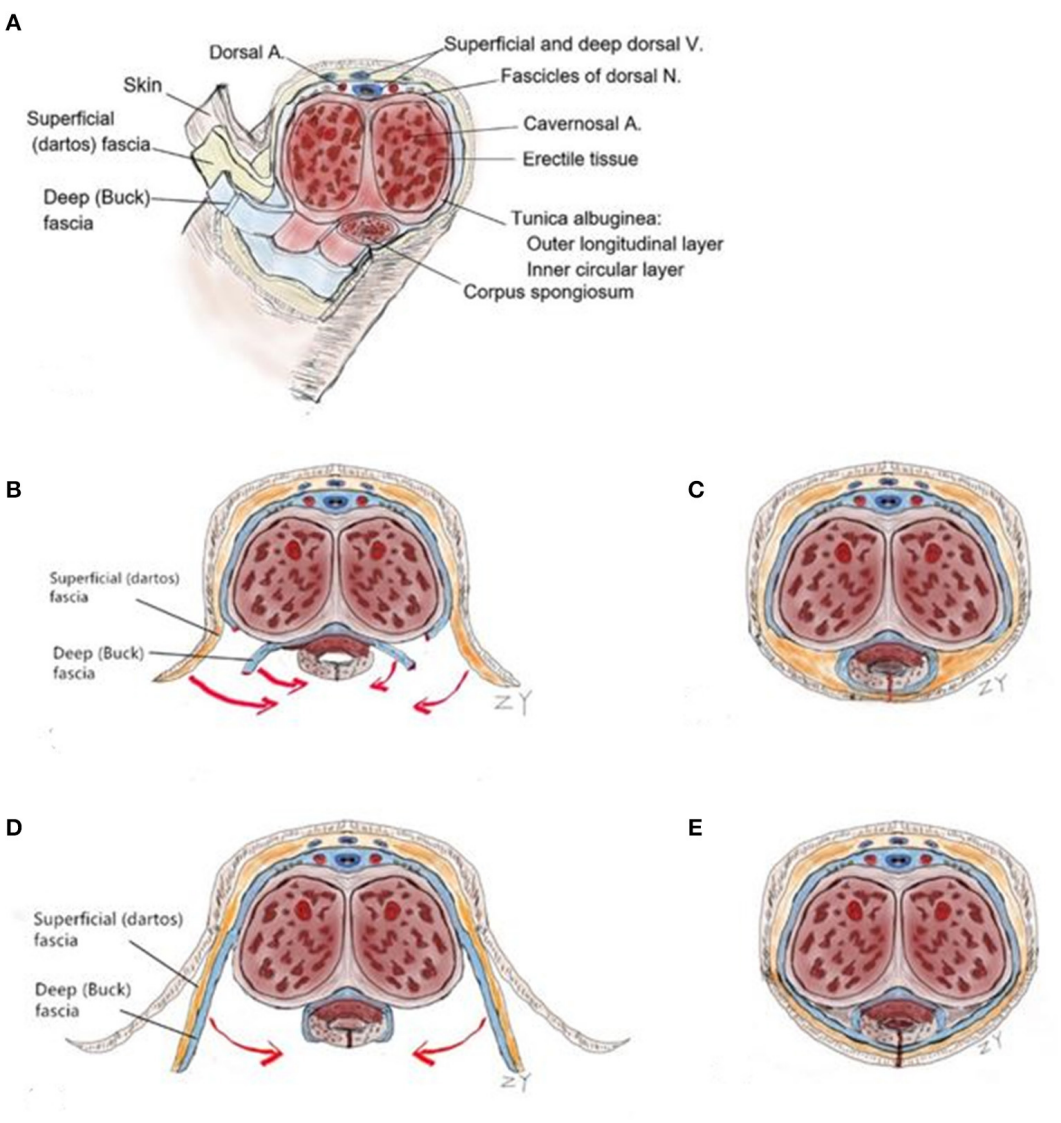
Diagrammatic sketch of the BFIC technique compared with the spongioplasty. (**A**) Top, a cross-section of the penis at the junction of its middle and distal thirds. In: Carson C, editor. Topics in clinical urology: complications of interventional techniques. New York: Igaku-Shoin; 1996. p. 86–94. (**B**) Incision of the general TIP procedure or Y-I spongioplasty. (**C**) The deep (Buck) fascia on the ventral side of the penis was interrupted post-operatively. (**D**) Incision with the BFIC technique: we cut the Buck's fascia close to both sides of the urethral plate, and then dissected laterally. (**E**) Buck's fascia completely covered the ventral side of the penis, emulating the normal anatomical structure.

DF and TVF is effective in protecting the neourethra as a covering for the suture line for many years during hypospadias repair. However, harvest of TVF may have the risk of damaging the vas deferens or vessels of the testicles, resulting in scrotal abscess or scrotal hematoma, and the dissection of DF may compromise the vascularity of the preputial skin covering and result in subsequent skin necrosis (16). Consequently, we are continuously seeking suitable methods and covering materials for urethral reconstruction. BF is the tough, elastic layer immediately adjacent to the TA (23, 24), and the penile shaft is surrounded by BF, DF, and skin. Distally, BF is attached to the undersurface of the glans at the corona (Figure 5A) (25). BFIC is not a type of spongioplasty, such as the Y–I spongioplasty (11), which creates rotation of the spongiosum by catching the fascia above the divided spongiosum (Figures 5B,C); it is also not a covering by the lateral BF. In BFIC, the BF is incised on both sides of the UP and separated from the TA along with both sides of the glans. The distinguishing feature of our BFIC method is the integration of the anatomy of the BF combined with the glans wings so as to restore the intermediate-layer structure of the penis and provide a strong support for covering the neourethra (Figures 5D,E). Baba et al. (26) applied BF as an intermediate layer in distal and proximal hypospadias with no or mild VC, and there was a statistically significant difference in the fistula occurrence (2.5% in the BF group vs. 12.5% in the dartos group), but in the report of Baba, the integrity of the glans wings and BF was interrupted at the coronal level. Herein, we cut the BF close to the edge of the UP, fully freeing both sides of the corpus cavernosus and, thus, achieving a tension-free suture in the middle of the ventrum. BFIC can also assist in approximating the glans to the midline, thus, reducing the tension of the neourethra and the glans.

It is generally accepted that adequate straightening of the VC is one of the most important goals in the treatment of hypospadias. At present, there are a few epidemiological investigations on penile curvature in hypospadias. Bandini et al. (27) reported their study of penile curvature, and the results showed that curvature (>10°) was identified and corrected in 90.4% among 303 hypospadias surgeries over 5 years, and concluded that < 1/10 of the patients did not require curvature correction. In our study, the proportion of VC correction was 82.4%, which was the highest in XMCH (94.2%). We considered that curvature must be objectively assessed and properly corrected, as identified by artificial erection after penile degloving, and is an essential component of the surgical procedure. The principal methods of curvature management include dorsal plication, chordee excision, and ventral lengthening in severe cases. Snodgrass and Bush (28) investigated 73 cases of postoperative complications of proximal hypospadias, showing an incidence of recurrent VC of 83%; in another study, he also introduced a method of ventral lengthening by using three transverse ventral corporotomies through the TA (29). In our study, we found that after removal of the ventral BF and glans from the TA, the tension on the ventral aspect of the penis could be redistributed, and this is helpful in the correction of penile curvature.

The BFIC technique was primarily used in the plate preservation procedures such as TIP, and the complication rate was 8.5–16.1% across centers in our records−8.3% in the primary operations and 23.3% in the re-operations; the fistula incidence of TIP was 4.9 and 10.9% in the primary and re-operations, respectively. As far as the TIP procedure was concerned, the neourethra came from the original UP, and the BFIC restored almost all of the anatomical structures of the penis and glans.

In our study, BFIC can be used in proximal hypospadias and severe curvature cases with transection of UP by free graft or free-tube graft urethroplasty. In fact, due to the blocking of the connection between the DF and the TA, free-graft application actually was a compromise, but the application of a free graft has ushered in a new era in hypospadias treatment. There is an ever-increasing number of reports regarding the application of free skin graft in urethroplasty (30, 31); however, the results have varied from 9 to 58% (32). Satisfactory outcomes of free-tube graft urethroplasty have been obtained in our study; the overall complication rate was 9.9%, but only one center used this technology on a large scale. This suggests that the choice of approach varies with surgeon preference, and the results need to be further evaluated by the application of more centers. With a favorable condition of the UP or mild VC, the urethra can then be formed by a partial graft (such as graft inlay or onlay), such that the neourethra is more in line with the original anatomical structures.

Reporting outcomes in hypospadias surgery remains a challenge, and there are some limitations to the present study. First, reporting outcomes of hypospadias surgery requires a long-term follow-up (2, 9, 10, 33). Our study consisted only of short- and medium-term observations after hypospadias surgery. In contrast to long-term follow-up, the results of postoperative complications may be biased and affect the judgment of the overall effects of the surgical methods. Second, the imperfections in data collection and the lack of a unified evaluation system were also major limitations to this study. Despite these shortcomings, this was a large-sample retrospective study that reflected the application of the BFIC technique at several large centers in China in recent years, and the results were generally positive.

## Conclusions

BFIC is a safe, feasible, and effective technique for the repair of hypospadias. It can be used to restore the normal anatomical structures of the outer layers of the penis, and be applied in distal to proximal hypospadias repair. As a compromise, concurrent free graft or free-tube graft urethroplasty needs to be used in some proximal hypospadias. Feedback received on its initial application shows that BFIC can reduce the incidence of postoperative complications of hypospadias. However, due to some limitations of the study, long-term follow-up, and unified and comprehensive evaluation criteria are needed for its ultimate and accurate evaluation.

## Data Availability Statement

The original contributions presented in the study are included in the article/Supplementary Material, further inquiries can be directed to the corresponding author/s.

## Author Contributions

The data were collected by MC, W-pZ, Y-mT, and H-cC in their respective centers. The data were analyzed by YZ, K-pZ, R-gL, and D-hL. Operations were performed by MC, W-pZ, Y-mT, and H-cC in each center. YZ wrote the manuscript and prepared the figures. X-sZ edited the manuscript. All authors contributed to the article and approved the submitted version.

## Conflict of Interest

The authors declare that the research was conducted in the absence of any commercial or financial relationships that could be construed as a potential conflict of interest.

## Publisher's Note

All claims expressed in this article are solely those of the authors and do not necessarily represent those of their affiliated organizations, or those of the publisher, the editors and the reviewers. Any product that may be evaluated in this article, or claim that may be made by its manufacturer, is not guaranteed or endorsed by the publisher.
